# Ethiopian Indigenous Traditional Fermented Beverage: The Role of the Microorganisms toward Nutritional and Safety Value of Fermented Beverage

**DOI:** 10.1155/2020/8891259

**Published:** 2020-12-12

**Authors:** Niguse Hotessa, Jedala Robe

**Affiliations:** Department of Biology, College of Natural and Computational Sciences, Bule Hora University, P.O. Box 144, Bule Hora, Ethiopia

## Abstract

Ethiopia is one of the countries where a wide variety of traditional fermented beverages are produced and consumed for a long time. Traditional fermented beverages are those which are indigenous to a particular area and have been developed by the people using age-old techniques from locally available raw materials. Some of Ethiopian indigenous traditional fermented beverages products are Cheka, Keribo, Borde, Areki, Tella, Shamita, Booka, and Korefe, in which fermentation is natural and involves mixed cultures of microbes. The most common fermenting microorganisms, lactic acid bacteria and yeast, are used as probiotics, for improvement of organoleptic properties, for provision of nutritional quality and biopreservative. The nature of beverage preparation in Ethiopia, traditional household processing, associated microorganisms with a fermented beverage, and their contribution toward improving the nutritional value and safety, the extent, and its prospect in supporting the livelihood of people in Ethiopia need concern. Therefore, in the future, to improve its quality, it is important to standardize the methods of beverage fermentation processes.

## 1. Introduction

Fermented beverages are an essential part of diets in all regions of the world and comprise about one-third of the worldwide consumption of food and 20–40% (by weight) of individual diets [1]. It constitutes a major part of the diet in all parts of the world in addition to their role in social functions [1, 2].

In Ethiopia, a wide range of indigenous fermented beverages is produced from different raw materials such as barley, maize, honey, and wheat [2]. Indigenous processing methods of Ethiopian fermented beverage are different from locality to locality or from product to product. Among the Ethiopian indigenous fermented beverages, Tella, Areki, Borde, Keribo, Shamita, Booka, and Cheka [2–5] are produced and consumed. The fermented beverage such as Teji, Tella, and Areki are considered alcoholic, whereas Cheka, Korefe, Shamita, Keribo, Borde, and Booka are considered as nonalcoholic beverages [6]. In Ethiopia, indigenous fermented beverages constitute a major portion of the diet of traditional Ethiopian homes, besides also consumed in different occasions such as holiday, wedding ceremony, and Iqub (a form of traditional revolving saving in which people voluntarily join a group and make a mandatory contribution every week or pay a month) [7]. Their relative cheapness has a selective effect by providing a cheap alternative for the low-income groups of consumers [8]. There are four main fermentation processes which are alcoholic, lactic acid, acetic acid, and alkali fermentation [1]. Alcoholic fermentation marks in the production of ethanol and yeasts are the major organisms. Lactic acid fermentation is produced by the presence of lactic acid bacteria (LAB). The other fermentation process is the acetic acid that is produced by *Acetobacter* species. Alkali fermentation frequently takes place through the fermentation of fish and seeds, popularly known as a condiment [1]. Most of the Ethiopian local fermented beverages are products of the acid-alcohol type of fermentation [9]. The nature of the preparation of fermented beverages in Ethiopia is not complex and does not require expensive equipment. The preparation of many local fermented beverages is still practiced at the household level under uncontrolled conditions, using rudimentary equipment such as empty oil vats and earthen vessels, and the handling and consumption often take place under conditions of poor hygiene [4]. As a result, the beverage becomes of poor quality with inconsistency and failure in most cases [5].

In Ethiopian indigenous fermented beverages, fermentation is natural and involves mixed cultures of microbes. Thus, some microbes may participate in parallel, while others act sequentially with an altering dominant biota during fermentation. The isolation of such microbes should not only be confined to dominant organisms but also should include other microbes found in lower numbers which might have an important function in the process. The sources of these microorganisms are usually raw ingredients, the traditional equipment used for the processes, and from back slopping using a small portion of beverage from a previous fermentation [10]. Initiation fermentation of most traditional fermented beverages may be undertaken by different groups of microorganisms as far as sufficient fermentable sugars/starches are available in the substrate [11].

The most common fermenting microorganisms are LAB and yeast [12].

In fermentation technology, microorganisms are used for the production of specific metabolite such as acids, alcohols, enzymes, antibiotics, and carbohydrate, upgrade bioavailability of micronutrients, removal of antinutrient factors [10], and also improve organoleptic properties of beverages [13]. These necessitate the understanding of the processes and contribution of microorganisms involved in preparing the beverages.

Even though traditional fermented beverages have a long history in Ethiopia, there is no writing culture in most of the Ethiopian regions. Some studies have been performed on traditional fermented beverages in Ethiopia, showing some aspects of these beverages. Therefore, the purpose of this paper was to review studies made by various researchers on Ethiopian traditional fermented beverages regarding processing methods, microorganisms, and its contribution toward the nutritional value and safety of fermented beverages.

## 2. Some Ethiopian Traditional Fermented Beverages

### 2.1. Cheka

Cheka is a bowl of cereal and vegetable-based fermented beverages which is consumed in southwestern parts of Ethiopia mainly in Dirashe and Konso. People of all ages including infants, pregnant, and lactating women drink Cheka [14]. Cheka is mainly prepared from cereals such as sorghum (*Sorghum bicolor*) and maize (*Zea mays*) and vegetables such as leaf cabbage (*Brassica* spp.), moringa (*Moringa stenoptella*), and decne (*Leptadenia hastata*). Also, root and leaf parts of taro are used.

The malt used for Cheka preparation can be prepared from a single or mixture of cereals that includes maize, sorghum, barley (*Hordeum vulgare*), and finger millet (*Eleusine coracana*).

**Figure 1:**
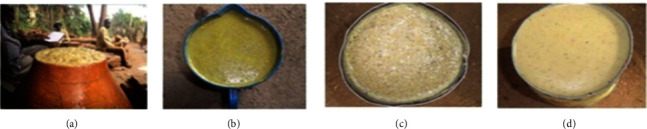
Cheka. (a) Fasha or chaqa and (b) hiba or parshota produced in low-land areas of Dirashe. (c) Hiba or parshota produced in high land areas of Dirashe and (d) menna (Photosource: Worku et al., 2015 [5]).

The processes of Cheka preparation are very complex and vary among households and localities. There are three types of Cheka produced in the Konso and Dirashe such as hiba, chaqa, and menna (Figure 1).

Most Cheka preparation methods involve three major phases that are marked by cooking [5, 12].

Phase I: in phase I, grain flour is thoroughly kneaded with water in “gebete” and allowed to ferment for 14 hours (for menna preparation) to over a month (in low-land rural areas of Dirashe). For home consumption and occasionally for sale, brewers in Konso use the leaves of taro to produce Cheka. In this case, taro leaves are chopped and cooked in a metal or clay pot. The overcooked taro leaves are allowed to ferment for about 6 days in a gebete. On the fourth day, the fermented product is mixed with a handful of malt and left to ferment for extra 2 days. Brewers believe that the added malt facilitates the decomposition of the leaves. After that, the fermented taro is mixed with fresh flour as usual and is kneaded with water which also ferments for 36–40 hours. This fermenting material is commonly referred to as pulota.

Phase II: the fermented product (pulota) is kneaded with little or no water and then made into dough balls called qabot (gafuma). The dough balls should not be less or much moistened. If the balls are less moistened, they become uncooked at the center and if too moistened, they are too tiresome for kneading. During cooking, pieces of dried hop wood or peeled barks of some plants are placed at the bottom of the pot or barrel and excess water is added to prevent the dough balls from burning. The balls are added when the water is boiled (93–95.5°C), and the barrel or pot is covered with a lid or a gourd that fits the pot. The dough balls are cooked for about 45 minutes to 1½ hours depending on the number of balls and intensity of the fire. The cooking of the dough balls in water would be expected to gelatinize cereal starch granules and, thereby, increase the efficiency of starch degradation by amylase [14]. The process of gelatinization occurs over a temperature range depending on the type and size of granules and starch to water ratio. Leaching of amylase occurs during gelatinization and thus creates available carbohydrates for the proliferation of fermentation microorganisms. Brewers often insert the stick into the balls to check whether they are cooked well or not. When the dough balls are cooked well, producers take one ball at a time and dip their hands quickly into the water in a container handled by the other hand to avoid damage to them. Then, the qabot is smashed in gebete using a beer bottle or a round-headed (pestle-like) material made from a wood called tomambayt. Once the dough balls are broken down into pieces, they are kneaded with little water and spread on a plastic sheet, large-sized gebete, or a bed made from wood to cool for few minutes to 7 hours.

After cooling, it is mixed with adequate milled malt, thoroughly kneaded, and allowed to ferment overnight in a gebete. However, most brewers in Dirashe allow this product to ferment for 36–40 hours to enhance the bitterness of the product. Most brewers spread a handful of malt on the surface of the kneaded product. The proportion of malt added during this phase can be as high as 25% of the unmalted ingredient [5]. The next day early in the morning, the product is transferred into a large fermentation vessel (barrel or rotto); water is added and is then well mixed together. This actively fermenting material is commonly referred to as sokatet (difdif). Sokatet can be stored for more than a week, and so, brewers may utilize a portion of it for preparing Cheka for home consumption and is usually given to respectable people and close relatives.

Phase III: on the same day, the Sokatet is transferred into large containers and mixed with water, and a very thick porridge (koldhumat or hanshalt) is prepared by pouring boiling water (94.5–97°C) on to flour in gebete and thorough mixing using a material made from wood for this purpose or a flat cattle bone (scapula). The porridge is allowed to cool to room temperature for 5–7 hours, and malt is kneaded with the cooled porridge. The amount of malt added at this stage depends on the strength of the sokatet and the amount of Cheka being produced. If the sokatet tastes much bitter, a small quantity of malt is added, or otherwise, it would increase [5]. Then, the koldhumat (equivalent term in Dirashe is hanshalt) is added into the vessel containing the sokatet; sufficient water is added and is thoroughly mixed together using a thick stick with a flat end. The Cheka is ready for consumption after 4–12 hours of fermentation. As the duration of fermentation in the preparation of hiba (Dirashe Cheka) is too long, the sokatet becomes much bitter, and as a result, the amount of malt added into hanshalt in the preparation of fasha (Konso Cheka) is slightly larger than for hiba, and also, the proportion of the sokatet in the final product is much greater than hanshalt in fasha. Even though Cheka is a popular fermented beverage in Konso and Dirashe, there is no scientifically documented information on the microbiology of Cheka.

The study by [14] reveals that the pH and titratable acidity of the samples ranged from 3.53 to 3.99 and 0.80%–1.11%, respectively. The crude protein, crude fat, total ash, and carbohydrate contents of the samples ranged from 3.12 g/100 g to 4.44 g/100 g, 1.17 g/100 g to 1.81 g/100 g, 0.65 g/100 g to 0.93 g/100 g, and 14.16 g/100 g to 19.03 g/100 g, respectively. The alcohol contents of the Cheka samples ranged from 3.04 to 8.96 (% v/v).

### 2.2. Borde

Borde is traditional fermented beverages. It is a popular meal replacement in Southern Ethiopia and western parts of the country [4]. Borde is considered to be a low-alcoholic beverage around 3.35 ± 0.64 (% v/v) mean value [15]. People believe that Borde enhances lactation, and mothers are encouraged to drink substantial amounts of it after giving birth [6]. It appears that the ingredients for Borde fermentation vary among Borde-producing communities. Maize was reported to be the major ingredient of Borde preparation in Southern Ethiopia, whereas in other areas such as Addis Abeba, wheat was the preferred ingredient. Other cereal ingredients such as sorghum (*Sorghum bicolor*), finger millet (*Eleusine coracana*), and tef (*Eragrostis tef*) are also used as ingredients. The major equipment used for the preparation of Borde are earthenware pots and griddle, grinding stones, bowls, and wonnfit (a sieve with a mesh of interwoven grass-fiber threads at the bottom) [7].

The barley is important for the preparation of malt. However, the processing steps are not markedly different. For malt preparation, barley is cleansed to remove dirt and extraneous materials and steeped in water for about a day. Excess water is drained off, and the soaked barley is allowed to germinate for five days wrapped in banana leaves. Later, germination barley can be sun-dried and ground finely.

**Figure 2:**
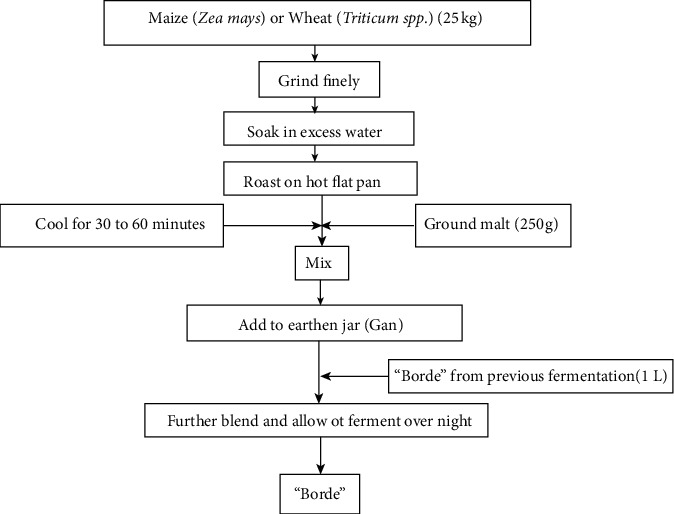
Flow chart of the Borde fermentation process [15].

The wheat/maize flour can be prepared and soaked in water. Then, the thick coarse paste is deeply roasted on a hot flat metal pan. After cooling one hour, ground malt is thoroughly mixed into it. The whole mixture is put into the earthen jar and further blended in tap water. The starter is added and sealed well with plastic films and cloth and allowed to ferment at ambient temperature for 24 hours (Figure 2).

The study conducted by [16] on microbiological and nutritional properties of ready-to-consume Borde in Awassa town reported that the mean pH value of Borde was reduced to 4.1 and counts of aerobic mesophilic bacteria and lactic acid bacteria were high (around 10^9^ CFU/ml). The counts of Enterobacteriaceae were around 10^6^ CFU/ml, whereas yeast count ranged between 10^7^ CFU/ml and 10^8^ CFU/ml for the product. Total protein, soluble protein, fat, ash, and carbohydrate content of Borde was 9.55%, 3.31%, 6.88%, 3.66%, and 8.88%, respectively, and compared with the raw ingredient, fermentation resulted in increased protein, fat, and ash contents of the finished product. The higher carbohydrate and protein contents of Borde justify people's claim for its use as a meal replacement among those who cannot afford reasonable meals for their daily activities. According to a study by [4], lactic acid bacteria had initial counts of 10^5^ CFU/ml and reached counts as high as 10^9^ CFU/ml after 24 hours. Hetero fermentative *lactobacilli* dominated the lactic flora throughout the fermentation, and a steady increase in yeast count was observed as the fermentation proceeded. The pH of fermenting Borde declined from 5.2 at the start to 3.8 at 24 hours [15].

### 2.3. Keribo

Keribo is an indigenous traditional fermented beverage and consumed in south, southwestern, and eastern part of Ethiopia. It is produced mainly from barely, honey, and sugar. Fermented Keribo serves in social functions such as on holidays, wedding ceremonies, and household consumption. About 30% of the population has used Keribo for income generation as well [12]. Keribo is a nonalcoholic beverage and popular among both adults and children as a drink and was consumed daily, especially during the dry season. The product has poor keeping quality with a shelf life of not more than 2 days. It should be preferably consumed 8–10 hours after fermentation without any refreshment with sugar [12].

**Figure 3:**
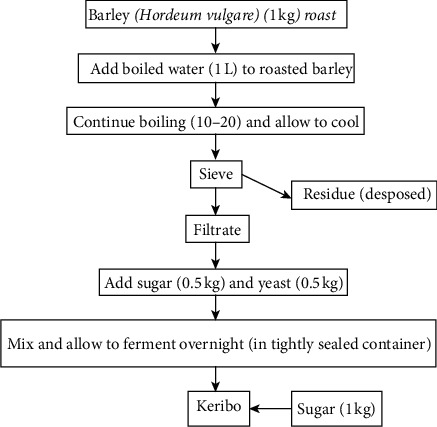
Flow chart of the traditional Keribo fermentation process [12].

For Keribo preparation, barely grain was first cleaned of broken kernels, chaff, and extraneous materials. Then, the deeply roasted barley is added to boiling water and continued boiling for 10–20 minutes at 65–75°C until the ungrounded grain seems to be dissolved. Finally, it is allowed to cool and sieved. Then, the boiled water-barley flour mixture was filtered using a wire mesh sieve. To the filtrate, sugar and yeast were added. Thereafter, the containers were closed with lids and left to ferment overnight and started to be served (Figure 3).

The study showed that the samples of Keribo from open markets and households in the Jimma zone, the average LAB, aerobic mesophilic bacteria (AMB), aerobic spore formers (ASF), and yeasts are with mean counts of 2.70 ± 2.07, 2.34 ± 2.37, 4.96 ± 2.80, and 4.96 ± 0.60 (log CFU/ml), respectively, after 6 hours of fermentation time. But the microbiology of Keribo samples drawn at an interval during controlled laboratory fermentation was observed to have mean counts of *Coliforms*, *Enterobacteriaceae*, *Enterococci*, and *Staphylococci* below the detection level. [12]. The initial high pH 5.75 of the Keribo fermentation at 0 h would explain the reason for the growth of aerobic mesophilic bacteria (AMB), while the lower pH (pH = 4.47) at 6 hours of fermentation began to inhibit their growth. The high numbers of LAB attained after 6 hours of fermentation were responsible for a marked reduction of pH and increment in titrable acidity resulting in inhibition of most aerobic mesophilic bacteria (AMB). The mean counts of yeasts increased throughout fermentation (for 48 hours) of the laboratory prepared Keribo. Likewise, there was an increase in the number of LAB and aerobic spore formers [17].

### 2.4. Tella

Tella is a popular Ethiopian traditional fermented beverage. The raw material is varying from place to place due to availability, cost, the test of final production, and other criteria. Some of the most common specified raw materials for most of Tella production in Ethiopia are barley, dagussa, sorghum, teff, and maize [9] in a different region. Depending on the type of cereal ingredients used to make, Tella has different names, and Amhara Tella has gesho (*Rhamnus prinoides*) and is concentrated. Gurage Tella is delicately aromatized with a variety of spices. Oromo Tella has no gesho (*Rhamnus prinoides*), and it is thick and sweet [9]. Tella varies in alcohol content usually around 8.1–14.59 (% v/v) [18].

For the preparation of Tella, the fermenter tank that is traditionally named earthen clay pot container (“Insera” in Amharic or “Gaanii” in Afaan Oromo) is washed with water and cleaned with leaves of “grawa” (*Vernonia amygdalina*) several times. Then, the cleaned container is inverted over smoking wood fragments of “weyra” (*Olea europaea*) for about 10–15 minutes. This will remove microorganisms that are sensitive to wood smoke and adds the desired flavor to the product [18].

The ingredient from which Tella is made, barley, is soaked for three to four days in water. As Debela et al. [18] explained in their finding, this may be important to reduce the energy/wood consumption during cooking, to prepare the sugar present in barley for enzymatic or microbial action, and to get equal distribution of heat during roasting. Then, “Bikil” (malt), the source of amylase from corn or barley or wheat grain germinate, is sun-dried. Then, it is roasted by firewood using biret mitad (metallic material used for roasting) until the color turns medially dark to attract customers.

Then, grinding of roasted barley, malt, and gesho (hop) to appropriate fraction for better hydrolysis and enzymatic action is carried out. Enkuro is prepared by adding water to roasted barley flour and heat treatment up to the color changes to near black for ease of fermentation. The color of Tella which may vary from light yellow to dark brown is determined by the extent of toasting enkuro [18]. Yetela kitta can be used for the same reason as enkuro but the preparation is different. Only the raw powdered barley was well mixed with a small proportion of water and can be agitated well. Then, after 1–3 hours of waiting, it can be baked. The baked bread was then dried and grounded into pieces to make the sugar ready for *S. cerevisia*e for conversion to alcohol [18].

The mixture of enkuro, the rest of the germinated grains (bikil), some gesho (hop), and water are added to the container. The importance of gesho (hop) is for the bitter and aromatic qualities that they lend to brewing [1]. The mixture is kept covered overnight, after which more water is added, and the container is kept sealed for 5–7 days until the beverage is ready to drink which can be kept even for 10–12 days without deterioration [1]. The only thing that happens in this step is a dilution of the highly concentrated Tella to commercialize one. At this step, two different products pure and residues are produced. The pure product serves for drinking purposes, while residues are sometimes diluted again and again to get pure less in quality Tella product. The pH value of Tella was 3.9, and the fermenting organisms composed of *Saccharomyces* spp., (mostly *Saccharomyces cerevisiae*) and *Lactobacillus* spp., (mostly *Lactobacillus pastori*um), *Acetobacter* spp., and *Bacillus* spp. after 12 hours of fermentation time (Table 1).

### 2.5. Shamita

Shamita is another traditional fermented beverage of Ethiopian, which is low in alcohol content, made by overnight fermentation of mainly roasted barley flour and consumed as a meal replacement. It has a thick consistency, and most people who cannot afford a reasonable meal consume it as a meal replacement [7].

**Figure 4:**
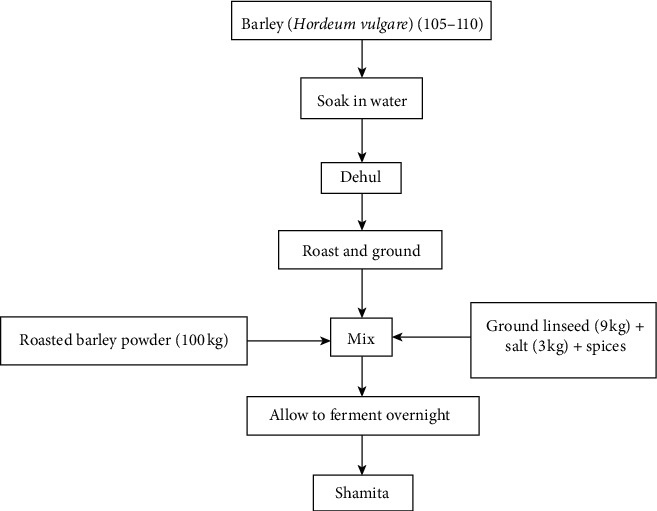
Flow chart of the Shamita production process by [2].

For Shamita preparation, lightly roasted barley is ground to which salt, ground linseed, and a small number of spices are added to it. Ground linseed is believed to ensure the thick consistency of the product. These are mixed with water, usually, in the evenings, and the product is ready for consumption in the morning (Figure 4).

The pH of Shamita was dropped from the initial value of 5.8–4.2, and the product had high microbial counts of lactic acid bacteria (6.1x10^9^ CFU/ml) within 24 hours of fermentation [16]. According to [19], the counts of Enterobacteriaceae were around 10^6^ CFU/ml, whereas the yeast count ranged between 10^7^ CFU/ml and 10^8^ CFU/ml at 24 hours fermentation time.

Negasi et al. [2] isolated potential probiotic lactic acid bacteria such as *Lactobacillus, Leuconostoc, Pediococcus*, and *Lactococcus.* These microorganisms could make the product a good source of microbial protein. Compared to a major ingredient, barely, Shamita had more total protein, fat, and ash values of 10.37%, 3.46%, and 6.85%, respectively [16].

### 2.6. Korefe

Korefe is the name of the indigenous traditional fermented beverage made in Begemder province among the Koumant ethnic group in Ethiopia. Dehusked barley is left in water overnight and after that toasted and milled. It is mixed with water and dried gesho leaves and fermented in a clay container for two to three months. When the beverage is needed, a small quantity of the mixture is taken, more water is added, and after a day's fermentation, the beverage is ready for consumption [7]. Yeasts are organisms that are responsible for the fermentation process of Korefe. The average alcoholic contents of Korefe ranged from 4.08% v/v to 5.44% v/v [7].

### 2.7. Areki

Areki is a colorless, clear, and traditional alcoholic beverage distilled from the fermented product. It is prepared in almost the same way as Tella except that the fermentation mass, in this case, is more concentrated [20]. Areki fermentation product is known as Yereki-tinsis which is prepared by mixing powdered gesho leaves and powdered bikil (1 :  2 ratios) with water to give a mixture of free-flowing consistency and will be put aside to ferment for about five days [9]. Traditionally Areki is classified as Terra-Areki and Dagim-Areki. Terra in Amharic refers to “ordinary.” An amount of dagussa (*Elusine coracann*) roughly equivalent to four times that of the bikil is powdered, kneaded with water to make dough, and baked into cakes. The hot cakes are broken into pieces and added to the first mixture, and with more water, it is well mixed, and again left aside to ferment for about four days. Portions of the second mixture are transferred to the traditional distillation apparatus and distilled to give what is known as Terra-Areki [9]. The term Dagim in Amharic refers to “second time” and designates that it is distilled the second time. It is a stronger type of Terra-Areki, which is prepared in the same way as Terra-Areki, except that the distillation process is allowed to proceed for a shorter period of time, or three volumes of Terra-Areki are redistilled to give about one volume of Dagim-Areki [9]. The alcohol content of Dagim-Areki has relatively high mean value, 48.00% (v/v), with a range of 38–48% (v/v), and even Terra-Areki has high mean alcohol content, 37.22% (v/v), with a range of 30.20–39.90% (v/v) [6], whereas the pH value of ready-to-consume Areki was at the range of 4.3–4.5 (Table 2) when it is considered to be the most suitable for consumption. Since the government has no control over the production of locally brewed alcoholic drinks, it is difficult to estimate the amount of alcohol production and consumption in Ethiopia.

### 2.8. Teji

Teji is yellow, sweet, effervescent, and cloudy containing residues of substrates. It is prepared from a mixture of honey and sugar, and also, leaves of gesho (*Rhamnus prenoides*) are added to give special flavor [6]. During the preparation of Teji, the fermentation pot is seasoned by smoking over the glowing of *Olea Africana* and gesho (*Rhamnus prinoides*). Honey, which may contain various impurities including wax, is mixed with water and placed in the smoked pot. The pot is covered and fermented continuously for five more days, in warmer weather (above 25°C), or for 15–20 days, in cooler environments (below 20°C) [9]. The mixture is stirred daily and finally filtered through cloth to remove sediments and *Rhamnus prenoides* and finally used for consumption. Teji produced in a different part of the country has a different flavor depending upon the nectar used in the production of honey and the sugar, and also, leaves of gesho (*Rhamnus prenoides*) are added to give special flavor [6]. The alcoholic contents of Teji were measured and found in the range of 8.94–13.16% v/v ethanol [9]. The study by [15] on microbial, physicochemical, and proximate analysis of selected Ethiopian traditional fermented beverages showed that LAB and yeast were the dominant microbes in Teji samples. Accordingly, the mean counts (log CFU ml^−1^) of LAB and yeast fall within the range of 6.09 ± 0.53–8.13 ± 0.67 and 6.31 ± 0.63–8.43 ± 0.72, respectively. The pH value of ready-to-consume Teji was at the range of 3.94–4.45. Mean value for total carbohydrates total lipids, total protein, and reducing sugars was 1.49–3.79 g/ml, <1 g/ml, 0.33–4.66 g/ml, and 0.46–2.09 g/ml, respectively [16]. The fermentation of Teji relies on the microorganisms (lactic acid bacteria and yeast) present in the substrates, fermentation vats, and equipment. Lactic acid bacteria are known to produce a variety of chemical compounds relative to fermentation conditions.

Their metabolic products contribute to acidity and also add distinctive flavor and aroma to the fermenting material. As mentioned somewhere else (Table 3), major yeast species in Teji were *Saccharomyces cerevisiae*, *Kluyveromyces bulgaricus*, *Debaromyces phaffi*, and *Kluyveromyces veronae,* whereas lactic flora consisted of *Lactobacillus*, *Streptococcus*, *Leuconostoc*, and *Pediococcus species*. Yeasts of *Saccharomyces* were reported to be responsible for the conversion of sugar to ethanol in Teji.

### 2.9. Booka

“Booka” is also an indigenous traditional fermented beverage in South Ethiopia, particularly consumed in Guji communities. Booka is the first animal origin traditional fermented beverage.

It is a liquid slightly yellowish made of “Booka” from cow bladder, so the product is named as Booka. Booka is found sometimes at the bottom of “Buttee” (a traditional instrument that is used to ferment milk).

However, the one that is used to ferment beverage (Booka) is usually from cow bladder. Equipment such as wooden bowl (Qorii), cup (Kookkii), container (gan), and filters are used, whereas ingredients such as honey, sugar (sometimes), Booka from cattle bladder, and water are used.

People of all ages including infants, pregnant, and lactating women drink Booka [23]. It had been consumed for ceremonies such as marriage, blessing (Eebbaa), Gadaa power transition (Baallii dabarsaa), and conflict resolution (Araara), “Gondooroo” and as a source of income generation. Gondooroo implies declaring or concluding something or an event that did not happen again. The “Gondooroo” tradition is performed not only as a mechanism of purifying the blasphemy from the guilty but also as a method of conflict resolution in Guji [23]. Indigenous production and preparation methods of Booka is not difficult; it can be prepared using ingredients such as honey, sugar (sometimes), and water and using Booka from certain types of cow bladders as an inoculum.

**Figure 5:**
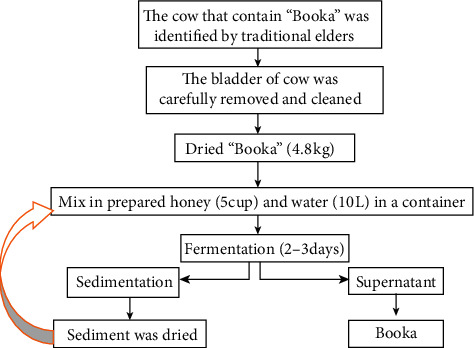
Flow chart of Booka production [23] by some modifications.

The Guji people (mostly older people) know which cows to be selected for this process, but the cause of the existence of the “Booka” in the bladder of a cow is unknown exactly. The liquid, the bladder of a cow is carefully removed, cleaned, and then filled with a honey and water solution in a container. Then, it will be enhanced (Ukkaananii kaasan) with pure honey and used immediately because the fertility of this “Booka” will be more fastened if this type of pure honey is used. But, if they need to preserve for a long period, they mix fresh Booka with honey and water with appropriate ratio and then dry, pack, and put for future usage. For immediate use, they inoculate this active Booka in honey and water in a container and store for two to three days, during which time it undergoes fermentation (Figure 5).

After fermentation is completed, the upper layer will then be ready to drink, while the bottom (sediment) will be reused again to ferment another Booka (beverage). Traditionally, the people can identify the production of Booka by its sound.

The popularity of this traditionally fermented beverage is more reflected among all age groups. However, there is no scientifically documented information on the microbiology of Booka. Therefore, Booka fermentation needs further investigation soon [23].

## 3. Substrates for Beverage Fermentation

In the preparation of wines from the starchy raw materials such as wheat, barley, rice, or corn, the raw materials must be degraded into sugars to ferment them by yeasts. Thus, traditionally fermented beverages throughout the world could be grouped into two main categories based on the types of substrates used for their preparation and production of ethyl alcohol. The first group of fermented alcoholic beverages in which sugars are the principal fermentable carbohydrates includes Ethiopian Teji [6] and others such as Indian jack fruit wine, Mexican pulque, and Kenyan urawaya [24]. The second principal fermentable carbohydrate is starch. For the fermentation process to occur, the starch should be hydrolyzed into simple sugars. Such hydrolysis could be achieved by malting (germination) or by using amylolytic molds and yeasts [25].

## 4. Role and Importance of Microorganisms in Fermented Beverage

### 4.1. Improvement of Organoleptic Properties

Microbial fermentation makes the fermented beverage palatable as there will be an improvement on the organoleptic properties, texture, aroma, and flavor [13]. Fermentation of Teji relies on the microorganisms (LAB and yeast), and their metabolic products contribute to acidity and also add distinctive flavor and aroma to the fermenting material [6]. LAB isolated from various fermented foods produces organic acids and a high diversity of antimicrobial agents, which are responsible for the upkeep of quality and the palatability of fermented foods. Yeasts of genus *Saccharomyces* were reported to be responsible for the conversion of sugars to ethanol in Teji. According to the finding by [18], after 10 days of fermentation, Tella becomes more acidic to consume due to the growth of *Acetobacter* spp., which converts ethanol to acetic acid under anaerobic conditions. The organoleptic properties of the fermented beverage make them more important, since it has wider acceptance [26].

### 4.2. Use as Probiotics

Probiotics are usually defined as microbial food supplements with beneficial effects on consumers. Probiotics have a great potential for improving nutrition, soothing intestinal disorders, improving the immune system, optimizing gut ecology, and promoting overall health because of their ability to compete with pathogens for adhesion sites, to antagonize pathogens, or to modulate the host's immune response, pharmaceutical preparations, and functional foods for the betterment of public health [20, 27]. In many communities around the world, there are traditional beliefs that some fermented foods or beverages have medicinal value. Hence, rural communities are known to be consuming fermented beverages/foods such as Borde [28], Booka [23], or other beverage/food laden with probiotic microbiota, from which they derive health benefits.

Most probiotic products contain LAB and molds that have been found to produce antibiotics and bacteriocins [28, 29]. The LAB belongs to the genera *Lactobacillus*, *Bifidobacterium*, *Enterococcus*, *Lactococcus*, *Streptococcus*, and *Leuconostoc* [28], and *Lactobacillus plantarum* strain CIP 103151, *Lactobacillus paracasei* strain NBRC 15889, and *Lactobacillus plantarum* strain JCM 1149 [29], inherently present in fermented Borde and Shamita, have antimicrobial properties against various foodborne pathogens invading the gastrointestinal tract. Thus, a better understanding of the intestinal microbial populations will contribute to the development of new strategies for the prevention and/or treatment of several diseases.

### 4.3. Provision of Nutritional Quality

Fermentation processes increase the digestibility and availability of nutrients [8]. The enzymes such as amylase, proteases, lipases, and phytates modify the primary food products through hydrolysis of polysaccharides, phytates, proteins, and lipids [26]. For instance, those beverages that use malt are known to contain much more free amino nitrogen than does the original grain, i.e, the partial degradation of reserve proteins in cereals makes the free amino nitrogen available [11]. The number of proteins and the content of the water-soluble vitamins increase, while the antinutrient factors (ANFs) in the foods decline during fermentation [13]. Palm wine in West Africa is high in vitamin B12, which is very important for people with low meat intake and for who subsist primarily on a vegetarian diet, idli (a LAB fermented product consumed in India) is high in thiamine and riboflavin as mentioned in [24].

Lactic acid fermentation of cereals has been used as a strategy to decrease the content of antinutrients, such as phytate and tannins [28]. This leads to increased bioavailability of micronutrients such as zinc, calcium, phosphorous iron, and amino acids. The high microbial load of yeast and lactic acid bacteria qualify Borde as a good source of microbial protein [9]. The mean crude protein content of fermented food increased from 0.74% to 4.58% (3 folds increment) after 48 hours of fermentation [17]. The highest levels of crude protein were also observed in fermentation samples with 1.5 ml inoculum of *Saccharomyces cerevisiae* for 48 hours [30]. The increase in the crude protein content was due to the effect of microbial cell growth [13].

### 4.4. Biopreservative Properties

The control/growth of one organism (undesired) by another has received much attention in recent years [31]. Many bacteria associated with fermented foods produce antimicrobial bioactive molecules, such as hydrogen peroxide, organic acids, and bacteriocins that make them effective biopreservatives. In fermented food series, biopreservation refers to the use/benefit of antagonistic microorganisms or their metabolic products to inhibit undesired microorganisms in fermented food products, thereby upgrading food safety and extending the shelf life of foods [31].

In a study by [7] on the microbial ecology of Borde and Shamita fermentation, the count of some of the dominant microorganisms such as bacteria of the genus *Bacillus*, *Lactobacillus*, *Staphylococcus*, *Micrococcus*, and members of Enterobacteriaceae and yeasts of the genus *Saccharomyces* was markedly increased; likewise, drop in pH of Borde from 5.2 to 3.6 and Shamita from 5.82 to 3.98 was observed [16]. The lowering of the pH value is due to the production of acid. Acid content plays an important role in alcoholic beverages for the preservation of beverages. The acid produced inhibits the growth of pathogenic microbes which can cause disease, thereby, prolonging the shelf life of fermented beverages. For example, LAB has antifungal activities. By doing this, the shelf life of fermented food can be prolonged. Acetic acid and propionic acid produced by LAB strains interact with cell membranes and cause intracellular acidification and protein denaturation [32]. With these properties, associate microorganisms inhibit both pathogen and nonpathogen microorganism growth in fermented beverages.

## 5. Prospects of Ethiopian Traditional Fermented Beverage

Based on the important role played by the traditional African fermented beverages, the consumers tend to recognize these beverages as types of food rather than just beverages [17]. Moreover, traditional fermentation processes are increasingly attracting the attention of scientists and policymakers as a vital part of food security strategies [19].

Traditional fermentation processes were developed largely as an art, rather than through scientific principles, and they are integrated into village life, commonly utilize locally available raw materials, inexpensive, and part of the culture of local consumers. However, there is no scientific protocol for the production of traditionally fermented beverages [33]. The fermentation period is chosen according to the brewer's judgment. Even with simple, small-scale fermentation, some physical aspects such as temperature, relative humidity, and level of agitation and aeration are often poorly controlled and production techniques are not standardized. There is no way to assure that a consistently uncontaminated environment for the fermentation, the unpredictable processing environment, the hygiene of handlers, equipment, and facilities are not evaluated. There is a need in particular to provide training as to good hygiene practices that prevent contamination and on how to improve fermentation efficiency to deliver consistently acceptable outputs in terms of quantity and quality. It may also be possible to determine and increase shelf life through training in appropriate technologies undertaking pasteurization and/or refrigeration, which stop the fermentation.

The lack of knowledge or information on the effect of heat treatment, ingredient, and fermentation on the nutritional quality of the final product questions the nutritional value of the final product, and this needs designing and optimizing operating condition for the production of the high-quality beverage. The alcohol health risk associated with high consumption causes a problem in the case of beverages containing high alcoholic content such as Areki. Thus, control in the production and supervision with the development of a comprehensive national alcohol policy is necessary.

The other issues that need consideration are the matter of identifying and increasing the efficiency of the starter culture in detoxifying some tannin and phytate, as well as acrylamide and cyanide, and improving nutritional quality in the final product. Therefore, new opportunities provided by biotechnology are opening up possibilities to improve or upgrade traditional small-scale processes and make better use of agricultural products. Fermentation may be the most simple and economical way of improving cereal nutritional value, sensory properties, and functional qualities available at the local community level. In general, the fermented beverage is a promise in feeding additional segments of the increasing Ethiopian population in the future.

## 6. Conclusion

The traditional beverage preparation is predominantly a household phenomenon in Ethiopia. Every household appears to process traditional beverage starting from raw ingredients to the final products. As can be observed in previous studies, different workers reported different values for the parameters they measured during the fermentation process. Microbiological and chemical variability in the various products could be attributed to the spontaneous fermentation, as this depends on the microbiota naturally present in the substrates, on utensils, and equipment used. Many microorganisms associated with fermented beverage are used as probiotics to enhance the sensory property of beverages, to produce antimicrobial bioactive molecules, such as hydrogen peroxide, organic acids, and bacteriocins that make them effective biopreservatives, and to produce nutraceuticals to create functional beverages with increased bioavailability of nutrients. Therefore, for developing countries such as Ethiopia, fermented beverages could become even more important in feeding additional segments of the increasing population in the future.

## Figures and Tables

**Table 1:** Changes in moisture content, pH, and microbial count occurring during the fermentation of Tella.

Phase	Fermentation time	pH	*Acetobacter* spp. (cfu/ml)	*Lactobacillus* spp. (cfu/ml)	*Saccharomyces* spp. (cfu/ml)	*Bacillus* spp. (cfu/ml)
I (0–4 days)	0	5.2	1 × 102	1 × 10^2^	—	1 × 10^2^
	1	5.2	2 × 102	4 × 10^2^	—	3 × 10^2^
	2	5.1	5 × 102	1 × 10^3^	—	3 × 10^2^
	3	5.0	6 × 102	4 × 10^3^	2 × 103	3 × 10^3^

II (4–6 days)	4	4.7	8 × 103	2 × 10^4^	6 × 105	4 × 10^5^
	5	4.6	2 × 104	1 × 10^3^	2 × 107	8 × 10^5^

III (6–8 days)	6	4.8	6 × 104	7 × 10^5^	6 × 107	1 × 10^6^

IV (8–12 days)	8	4.6	5 × 105	3 × 10^6^	9 × 107	8 × 104
	10	4.5	5 × 106	2 × 10^7^	9 × 107	4 × 10^2^
	12	3.9	8 × 107	**7** × 10^6^	9 × 106	1 × 102

CFU, colony forming units. ^*∗*^*Acetobacter xylinum* was the most predominant spp. ^∗∗^*Lactobacillus pastorium* was the most abundant species. ^∗∗∗^*Saccharomyces cerevisiae* was the most abundant species. Source: Debela et al. [18].

**Table 2:** Experimental results of pH and ethanol level of Ethiopian traditional alcoholic drinks, Areki.

S/N	Collection area	pH	Alcoholic content (% v/v)
1	Qochi	4.30	36.99
2	Ajjip	4.40	33.95
3	Matrik	4.51	39.90
4	Markato	4.49	38.96
5	Menihara	4.48	36.30
6	Average	4.436	37.22

Source: Chandravanshi et al. [6].

**Table 3:** The main microorganisms associated with some of traditional fermented beverages.

Product	Types of microorganism			References
	LAB and other bacteria	Yeast	Mold	
Borde	*Enterobacteriaceae*, *Leuconostoc mesenteroid*, *Bacillus*, *Micrococcus*, *Streptococcus*, *Weissella confusa*, *Lactobacillus brevis*, *Lactobacillus viridescens*, *Pediococcus pentosaceus*, *Lactobacillus curvatus*, *Lactobacillus collinoides*, *Lactobacillus sanfrancisco*, *Lactobacillus pontis*, and *Lactobacillus delbrueckii* subsp.	Yeast (*Saccharomyces*) and *Rhodoturula* spp.	Mold	[3, 4, 8]
Keribo	*Leuconostoc mesenteric* and *Aerobic mesophilic* bacteria	Yeast	Mold	[12]
Teji	*Lactobacillus* sp. (mainly *Lactobacillus plantarum*), *Leuconostoc*, *Pediococcus* sp., and *Streptococcus*	*S. cereviseae*, *Debaromyces phaffi*, *Kluyveromyces bulgaricus,* and *Kluyveromyces veronae*	—	[6, 11, 21, 22]
Korefe	—	Yeast	—	[7]
Shamita	*Bacillus* spp, *Micrococci*, *Lactobacillus*, *Leuconostoc*, *Pediococcus* and *Lactococcus*, *Staphylococcus*, *Streptococci*, and *Staphylococci*	*Saccharomyces* and *Rhodoturula* spp.	Molds	[1–3, 8, 9]
